# Morgellons Disease Treated as a Psychosomatic Condition

**DOI:** 10.7759/cureus.25236

**Published:** 2022-05-23

**Authors:** Hunter A Cutlip, Raja Mogallapu, Michael Ang-Rabanes

**Affiliations:** 1 Psychiatry, West Virginia University School of Medicine, Martinsburg, USA

**Keywords:** delusional parasitosis, psychosomatic symptoms, psychosomatic disorders, chronic lyme disease, morgellons disease

## Abstract

This case report details the presentation of a patient who presented to Psychiatry via the Emergency Department following a diphenhydramine overdose in an attempt to seek relief from a perceived skin condition. Review of the patient's files demonstrated similar presentations to a number of other specialties including Family Medicine and Dermatology. Due to the description by the patient of his condition as well as the associated psychiatric symptoms and significant impairment to his life, he was diagnosed with Morgellons disease. This paper seeks to highlight the lack of physical findings by other specialties, elaborate on the treatment plan during his inpatient stay, and review the evidence regarding the psychosomatic presentation of the disease.

## Introduction

Initially described in 2002, Morgellons disease (MD) has a short but complicated history. Biologist Mary Leitao was the first to report the condition following an investigation into her son’s scabies diagnosis [1]. MD is complicated by inconsistent skin findings, comorbid psychiatric conditions, and a potential association with spirochetal infection [2]. The Diagnostic and Statistical Manual (DSM) does not recognize MD and therefore has no concrete diagnostic criteria for diagnosis or treatment. In this case report, we describe a patient with an acute onset of delusional parasitosis, further classified as Morgellons disease, and treated with risperidone.

## Case presentation

A 45-year-old male with a history including anxiety, depression, peripheral neuropathy, and suspected Morgellons disease presented to the emergency department complaining of a several-month history of a rash as well as an ongoing concern for “little barbs” in his skin. He stated that the sensation occurred mostly in his face, arms, and upper torso; describing it as “crystallized threads” or “worms”. He requested a psychiatric evaluation and stated “I’d rather be dead than feel like this”. The urine drug screen was positive for methadone and cannabis. The provider in the emergency department placed the patient on a one-to-one observation until his mental status could be assessed and psychiatry was consulted, although a specific rationale was not noted.

The patient was admitted and one-to-one status was discontinued after an assessment revealed no active suicidal ideation (SI) or plan. During his intake interview, the patient explained that his symptoms began as stinging sensations in his scalp. Approximately a month after the onset of these symptoms without relief he felt that a shower he had taken in an attempt to reduce symptoms had led to the causative agent “entering [his] pores and spreading throughout [his] body”. After his shower, he noticed “white, rice-like strands” in his skin that he could express. He presented to the ED because he was having the “worst ever” attack he had experienced, with the sensation spreading into his mouth and nose. His wife reported that lately he had been taking exceptionally long showers, spending excessive amounts of time washing his hands in the sink, and compulsively picking and squeezing at his own skin.

Further elaboration from the patient on his observations about the cause of his symptoms revealed a complex belief system. He avoided the bathrooms and front door of his house as much as possible as he felt that these locations caused the “threads” to grow quicker. He stated that the cold also made them grow quicker. He felt that these threads left behind an “oily substance”. He can see them when he looks in the mirror but has yet to have any examiner find them. His primary concern regarding the “threads” is that they “would get too long and pierce one of my organs”. Prior to presenting to the ED, the patient’s symptoms had worsened such that he and his wife rented a hotel room so that he could get out of the house to escape the severe “attack” he was experiencing.

A review of the patient’s records demonstrated no evidence of previous parasite infection or Lyme disease. The patient had previously presented to several emergency departments on multiple occasions with these skin complaints, but had not received a tentative diagnosis until a hospitalization four months prior to the events of this case report. He initially presented earlier on the day of his hospitalization with nonspecific skin concerns, was released from the ED, and returned later after overdosing on diphenhydramine. He was obtunded at that time and accompanied by his wife. The patient spoke to the hospitalist after becoming more alert and stated that he had taken the medication in an attempt to soothe the burning sensation in his skin. Following this conversation, the hospitalist considered a diagnosis of Morgellons disease likely. The patient was evaluated by telepsychiatry who determined that the patient had no SI and that the overdose was accidental. The patient expressed his intention to check out against medical advice the following day preventing further workup or evaluation. The following week, the patient met with a dermatologist. He was given the preliminary diagnosis of delusion of parasitosis and had a skin biopsy taken to confirm that there was no pathologic process occurring. The skin biopsy was negative and the dermatologist contacted the patient to inform him of the results and recommend sensitive skin lotions and soaps as well as psychiatric evaluation. This conversation, in addition to the worsening of his symptoms, led to his presentation to the emergency department for the admission relevant to this article. Differential diagnosis included Morgellons disease and delusional parasitosis, but additional evaluation was required in order to differentiate these conditions.

The patient reported reduced irritation and dermatological sensation the next morning, but attributed his reduced symptoms to being out of his house. A polymerase chain reaction test for Lyme disease was negative. He denied any psychiatric symptoms, but was noted to have pressured speech and hyperfixation on his skin symptoms. He did not feel that he required psychiatric admission but was willing to stay while his wife cleaned their entire house “with Lysol and everything” in order to make him more comfortable returning home. He agreed to begin a 1mg dose of risperidone. He reported a significant reduction in physical symptoms and demonstrated a lower level of the associated fixation over the course of his stay. The patient would later endorse cannabis use recreationally “once in a while”, but refused to elaborate on dose or frequency. He decided to continue his treatment on an outpatient basis, and requested a family meeting in order to discuss his diagnosis and management with his family. He was discharged in less than a week and was lost to follow-up presumably due to seeking treatment with a psychiatrist outside of the hospital system as discussed during discharge, but the review of hospital records indicates that the patient has not sought treatment for skin conditions in this hospital system for over a year since discharge.

## Discussion

MD is clinically considered to be a psychosomatic condition; though it is not specifically referenced in the DSM. Previous studies have noted that patients diagnosed with MD tend to have other underlying psychiatric conditions such as depression and anxiety [3,4]. In the absence of a secondary causative factor such as intoxication or schizophrenia, MD is most similar to delusional disorder, somatic type [5]. Per this definition, a patient with MD is an otherwise rational individual who is experiencing the effects of a parasitic infection which often cannot be clinically observed. Although a schizophrenia spectrum disorder could reasonably be suspected, patients such as ours may not meet the full criteria to be diagnosed.

The nature of the somatic symptoms in MD is dependent on the source of the fibers or threads that are often found in the skin of patients [6]. Those who consider MD psychosomatic explain these fibers as being unintentionally self-implanted by patients who are constantly picking and scratching at their skin. Proponents of MD as a legitimate somatic condition argue that it is instead a long-term sequela of a spirochetal infection, most often Borrelia burgdorferi. One proposed method for the formation of these fibers involved keratin and collagen-based fibers resulting from dysregulated keratinocytes and fibroblasts [6].

Histopathology of MD patients does not demonstrate consistent spirochetal findings required for this to be considered a diagnostic feature of the disease. A CDC-backed investigation into the skin findings of 115 MD patients demonstrated no parasites on biopsy [2]. The study noted no discrete pattern in order, intensity, or onset of skin symptoms. Additionally, in patients whose biopsies demonstrated threads or fibers (43% of lesions), all of them were composed of substances that most likely originated from everyday items such as clothes, furniture, etc. All but one of these samples were of cellulose (likely plant fibers from cotton). Silicone, the final sample, could not be created organically. Finally, the study could not find a common infectious etiology between the patients involved, and their parasitology workup was unremarkable compared to a random sample from the population [2]. Case reports suggesting the efficacy of doxycycline in the treatment of MD have recently been published, however, these cases are associated with recent tick bites or Lyme disease for which doxycycline is the accepted standard of care [7].

Publications attempting to link Lyme disease to MD discuss common symptoms such as fatigue, joint pain, and neuropathy [6]. However, these symptoms are particularly non-specific in nature and can easily be explained by other conditions. For instance, fatigue and joint pain are common somatic manifestations of depression [8]. More specific indicators of inadequately treated or untreated Lyme disease, such as heart block or Bell’s Palsy, are not often cited in MD patients.

Treatment of MD requires a multi-disciplinary approach among dermatologists, psychiatrists, and primary care physicians. While medical management of underlying conditions is paramount in MD patients, it is equally necessary to provide therapy aimed at teaching the patient to manage their emotions. A self-reported questionnaire of current MD patients demonstrated highly prevalent levels of anxiety (52%), depression (41.6%), and overall poor quality of life (81%) associated with their perception of pain [3].

Some medications have shown promising results in case reports for the treatment of MD. While the inconsistent definition of the condition creates some difficulty demonstrating efficacy in drug trials, antipsychotics such as olanzapine have been shown to decrease somatic symptoms in Morgellons patients [9]. Similar studies have been performed using pimozide, but the significant side effects associated with the drug should be considered when initiating treatment [10,11].

## Conclusions

Despite some objections and disease models prominent among the Morgellons disease community, current evidence suggests that MD is most likely a primarily psychosomatic condition. The evidence linking Borrelia infections and fiber formation to patients diagnosed with MD is tenuous and not prevalent in enough cases to justify a causative relationship. Appropriate assessment of the patient’s full clinical picture with respect to underlying factors responsible for the patient’s presentation is paramount for patient improvement. This case report demonstrates a patient who was appropriately identified as having delusional features and was successfully treated with a combination of antipsychotic medication and psychotherapy.
